# Differences in the Development of Motor Skills in Portuguese Children Aged 12 Months after 3 Years of COVID-19 Confinement

**DOI:** 10.3390/children11080918

**Published:** 2024-07-30

**Authors:** Miguel Rebelo, Rui Paulo, Samuel Honório, João Petrica, Marco Batista, Pedro Duarte-Mendes, Catarina Marques, João Serrano

**Affiliations:** 1 Department of Sports and Well-Being, Polytechnic Institute of Castelo Branco, 6000-266 Castelo Branco, Portugal; ruipaulo@ipcb.pt (R.P.); samuelhonorio@ipcb.pt (S.H.); j.petrica@ipcb.pt (J.P.); marco.batista@ipcb.pt (M.B.); pedromendes@ipcb.pt (P.D.-M.); j.serrano@ipcb.pt (J.S.); 2 SPRINT Sport Physical Activity and Health Research & Innovation Center, 6000-266 Castelo Branco, Portugal; catarinam.14@hotmail.com

**Keywords:** COVID-19, motor skills, PDMS-2, children

## Abstract

(1) Background: The objective of the study was to verify the effects of COVID-19 confinement on motor skills through a longitudinal study in Portuguese children who were one year old at the beginning of the pandemic. (2) Methods: The sample consisted of 88 children of both sexes, in the pre-COVID-19 assessment, they were 13.31 ± 2.4 months old and in the post-COVID-19 assessment, the same children were already 49.31 ± 2.5 months old. Motor skills were assessed using the PDMS-2 scales. For the statistical analysis, the Kolmogorov–Smirnov test was used to test normality, and the Wilcoxon test was used to compare the results of the two assessments in the same sample. (3) Results: There were statistically significant differences in all motor skills assessed, with children presenting, on average, worse results in all global motor skills in the post-COVID-19 assessment, as opposed to fine motor skills, showing better results in the post-COVID-19 assessment. (4) Conclusions: These results show the negative impact of the pandemic on children evaluated with a special emphasis on global motor skills, with the majority demonstrating values considered below average for their age, noting that the pandemic protocols may have had serious consequences on children’s motor development, warning professionals who deal daily with children in these age groups about the importance of stimulating global motor skills.

## 1. Introduction

The emergence of the new coronavirus infectious disease that began in China in December 2019 caused a state of a Public Health Emergency of International Concern decreed by the World Health Organization (WHO) on 30 January 2020. However, it was only in March 2020 that the COVID-19 outbreak was characterised as a pandemic, spreading across different regions in the world.

The COVID-19 pandemic has caused a series of changes in children’s daily routines, impacting processes associated with child development [1]. It constituted the worst public health crisis of the century, disrupting daily life with the closure of daycares, preschools, and schools and, during lockdowns, isolated families in their homes with little outside support [2].

Most scientific evidence has suggested that the COVID-19 pandemic has negatively impacted student learning, disrupting the normal functioning of schools and leading to learning losses. It disrupted the normal functioning of schools and led to learning losses that have yet to be assessed, with the potential to increase inequality in education [3,4,5].

The Portuguese school system officially closed on 16 March 2020, during the first wave of COVID-19, transitioning to a mixed system of television broadcasting and online home teaching by June 2020 [6]. This abrupt change required rapid adaptation on the part of both teachers and students, who had to face a number of unprecedented technical and pedagogical challenges [1]. The second lockdown in Portugal took place from January to 15 March 2021 [7], and online teaching once again returned to the daily lives of teachers and students, but this time with higher levels of preparation for primary and secondary education, including improvements to online teaching platforms and greater technical support. On the other hand, and due to its specificities, education in nurseries and pre-schools had been completely neglected [1], significantly reducing the interaction between the school and the child, with children being referred to their parents without proper monitoring of their state of development, since children of daycare age (who were the case in our sample) were in confinement between 2 March and 17 March 2020 without any type of access to learning resources (unlike the other levels of education). On 18 March 2020, when they were able to return to their school environment, they had very strict learning rules with specific and delimited spaces for each child, without toys and without contact with other children and with the mandatory use of masks by educators. The return to daycare centres only lasted until October 2020, as on that date, daycare centres in this geographical area closed again due to an increase in cases per inhabitant until 3 May 2021, leaving children once again isolated in their homes. From 3 May 2021, they were able to return to daycare centres, still with all the previous restrictions, and only from 17 February 2022, they were able to return to their normal routines they had before the pandemic. This lack of educational support for children has raised concerns about the long-term impacts on their social and cognitive development.

From the outset, we heard warnings from experts that the closure of schools, daycares, and preschools, as well as the suspension of various sporting activities and the restriction of outdoor exercise, would have a negative impact on the physical (and mental) condition of the population, including children [8,9]. In particular, the closure of daycares would have a negative impact on children’s motor skills [8], compromising their ability to move (such as running, jumping, climbing, descending, etc.) and manipulate objects (such as throwing, catching, kicking, etc.). In this regard, the first reports on the impact of these lockdowns showed a decline in children’s motor skills and physical fitness after the first wave of the pandemic in 2020 [10]. In addition, the proposed measures eliminated the possibility of regular contact with other children and deprived babies of an essential part of the stimuli necessary for their development [11]. In this sense, studies on babies’ abilities before and after the pandemic are non-existent.

Knowing that children in the first years of life develop through their senses and stimuli from the surrounding environment [12], with confinement inhibiting contact with children and family and prohibiting the use of playgrounds and recreational spaces on the part of the entire population, these stimuli remained closed in each child’s home under the influence of parental involvement. It is also known that the socio-economic statuses of families had an impact on stimulation and learning during the pandemic [1]. Homes, particularly in Portugal, are spaces with increasingly smaller rooms, clearly limiting children’s mobility and play, favouring video games and digital activities even between parents and children [13]. Even before the start of COVID-19, a survey showed that 47% of parents of children aged four to thirteen played digital games together more often than outdoor games [14].

Although physical inactivity is known to be associated with obesity and delays in motor skills [15], there is a lack of information about the impact of the pandemic protocols on babies. In February 2020, while data were being collected to assess motor skills in 12-month-old children, we were faced with COVID-19 and the consequent isolation and confinement; however, the data already collected before the pandemic served as a starting point and opportunity to check the motor skills of the same children after three years and thus understand the impact of COVID-19 isolation on the motor development of children of these ages. Therefore, the objective of this study was to verify the effects of confinement on motor skills through a longitudinal study in children who were one year old at the beginning of the pandemic.

We thus intended to understand the impact of the measures imposed on children in these age groups and therefore raise awareness of the importance of more and greater motor stimuli after this confinement.

## 2. Materials and Methods

### 2.1. Participants

This longitudinal study involved 88 children (F = 35; M = 53). At the first evaluation (pre-COVID-19, February 2020), the children were 13.31 ± 2.4 months old, and at the second evaluation (post-COVID-19, February 2023), they were 49.31 ± 2.5 months old.

All these children lived in the urban environment of a city in Portugal, and none of them practised any type of physical activity before or after the lockdown. When their parents were asked what kind of activities they did during the pandemic, the answers were that play was limited to the everyday use of toys at home.

For the first data collection, contact had been established with the daycare centre to evaluate the children; subsequently, the same authorisation was requested for the second evaluation. Of the ninety-three children evaluated pre-COVID-19, only eighty-eight were still in the same institution and with the same educators after the pandemic, and only these were re-evaluated.

The following exclusion criteria were considered: children who did not belong to the initial sample of the first assessment before COVID-19, children diagnosed with learning difficulties and/or developmental changes, and children with some type of diagnosed disability.

### 2.2. Instruments

The scales from the Peabody Developmental Motor Scales–Second Edition (PDMS-2) [16] were used to evaluate children’s motor skills. The PDMS-2 are one of the most accurate and valid instruments for assessing motor skills in children from birth to 6 years of age. They were reviewed by Saraiva et al. [17] and Rebelo et al. [18] for the Portuguese population (χ2 = 55.614; df = 4; *p* = 0.06; χ2/df = 13.904; SRMR= 0.065; CFI = 0.99, TLI = 0.99; α = 0.85, and ICC = 0.98).

The results of the PDMS-2 are indicated in three domains of motor development: the global motor quotient (GMQ), which encompasses postural, locomotion and object manipulation skills; the fine motor quotient (FMQ), which encompasses the skills of fine motor and visuo-motor integration; and the total motor quotient (TMQ) that results from the previous two. With the results of each subtest, we were able to understand whether the child had not yet acquired a certain motor skill, and with the combination of all these factors, it was possible to indicate the motor profile of each child [17].

The items were summed up in each of the tests, and their values are located in the reference table for age, resulting in a standardised value and a percentile value that can be compared between ages. Finally, the standardised values can be converted into a qualitative classification with categories (17–20 Very Good; 15–16 Good; 13–14 Above Average; 8–12 Average; 6–7 Below Average; 4–5 Weak; 1–3 Very Weak) [16]. The scales are standardised for the child population and have a mean value of 10 points (±3) for each test [16].

### 2.3. Procedures

After approval by the Ethics Committee and the institution for data collection, the Free and Informed Consent Form was sent to the parents who were asked to fill out the child’s anamnesis form, which allowed the children to be selected, considering the inclusion and exclusion criteria of the study. All ethical principles, norms, and international standards relating to the Declaration of Helsinki and the Convention on Human Rights and Biomedicine were followed.

According to Folio and Fewell [16], all examiners who use the PDMS-2 must understand the general procedures for applying the test, its interpretation, and classification. In this research, only a single researcher, a specialist in the area of motor development and familiar with the instruments, collected the data.

The administration of PDMS-2 lasts approximately 45 to 60 min, individually and in a room with ample space, with the least possible amount of distracting materials that interfere with the assessment and with stairs nearby, with all routines of the daycare centre itself being respected. When interrupted, the assessments were completed within a maximum period of 5 days, as defined by the scale’s authors [16].

The following rules were followed for the correct application of the instrument: to give the opportunity to reach the maximum score on each item, the instructions were repeated three times for each child; the child started the test at a point on the scale established by their age (these points were empirically determined to allow the examiner to start the test on an item that 75% of the children in the normative sample of that age passed), proceeding in the sequence until the test is failed on three consecutive items. The score for each item is 0 to 2 (0 does not perform, 1 performs with difficulty, and 2 performs well) [17]. After the evaluation, the sum of each item is calculated until the final result is established for global, fine, and total motor skills (which is the sum of global and fine skills). Subsequently, the value of the sum of the items in each of the subscales is located in a reference table for age, where a standardised value is obtained (from 1 to 20), which can be converted into a qualitative classification with seven categories (from “Very Good” to “Very Weak”), with values between 8 and 10 being considered “average” for age [16]. These standard scores were used to compare the results in the two different assessment phases since, as the authors state [16], the PDMS-2 standard scores are the most effective way of assessing motor skills over time, because the assessment exercises differ according to age, but are subsequently standardised for the child’s specific age in a reference table and can thus be compared over time.

### 2.4. Statistical Analysis

To process the data, IBM–SPSS–Statistical Package for the Social Sciences SPSS (v.23.0) was used. Initially, the normality of the sample was checked with the Kolmogorov–Smirnov test, and as a non-normal distribution was obtained for all variables under study, the Wilcoxon test was used to compare the results of the two evaluations for the same sample. The method of inferences based on the magnitude of the effects was also performed, using the following scale (d Cohen): 0–0.2, trivial; 0.21–0.6, low; 0.61–1.2, moderate; 1.21–2.0, high; >2.0, very high [19].

## 3. Results and Discussion

Table 1 presents the results of the comparative analysis of the motor skills of the same children between the first assessment before the COVID-19 pandemic (pre-COVID-19) and after the pandemic (post-COVID-19).

We can observe that there were statistically significant differences in all global motor skills (postural, locomotion, and object manipulation) and in fine motor skills.

For global motor skills, before the pandemic, the children showed normative values considered to be “average” for their age. On the other hand, after the pandemic, especially for locomotion skills (7.44 ± 0.82) and object manipulation (7.15 ± 1.96), they had lower normative values and were considered “below average” for their age.

In terms of fine motor skills, these same children showed better results and abilities after the pandemic (post-COVID-19), particularly in visual-motor integration (11.97 ± 1.19), where they showed above-average values for their age. Since the lockdown gave them greater access to mobile phones, tablets, video games, and televisions, this could be the main reason why visuo-motor integration improved. On the other hand, global motor skills tended not to evolve, with some logic, since the children were deprived of contact with the outside environment and nature and were left to rely solely on their living space to develop fundamental global motor skills such as walking, running, jumping, kicking, and throwing.

Also, analysing the Effect Size (η2—Eta squared), we verified a very high effect for postural and object manipulation skills, a high effect for locomotion and fine motor skills, and lower effects for visuo-motor integration skills.

The results for global motor skills clearly indicated that changes in daily routines, stimuli, and children’s experiences with confinement and isolation were harmful to their development process [8]. Neuroscience states that until the age of two, the child’s brain develops at a unique speed and can make around 1,000,000 new neuronal connections per second [20]. These connections allow the child to develop emotional, cognitive, and social skills. Given this, it is essential to create an environment that encourages the development of these skills, especially for children in this age group, preventing their development from being compromised. Corroborating this point of view, Bonow et al. [21] stated in their study that mothers of babies born and raised during the pandemic were concerned about the socio-affective and motor development of their children due to social isolation.

Silva et al. [22], on the other hand, showed that children after confinement showed changes in behaviour, bringing with them signs and symptoms of anxiety, fear, agitation, stress, and insomnia, these factors being associated with the lack of pleasurable activities such as going to school and playing with friends, thus not having the possibility of spending all their energy at home on a daily basis, which is necessary and fundamental at these ages, and therefore they lacked a greater number of stimuli to improve some fundamental motor skills. For their part, Ferrari et al. [23] and Silva et al. [24] referred to the positive aspects of children spending more time with their parents, in some cases favouring the family environment.

The results verified for global motor skills clearly indicated that changes in daily routines, stimuli, and children’s experiences with confinement and isolation were harmful to their development process [1,8], particularly motor development, as mentioned in the study by González et al. [24] who state that in addition to children’s cognitive development, motor development was also what suffered most during this pandemic period. Studies with other ages and samples from different nationalities, namely French [25], Austrian [15], and Slovenian [26], also found a decline in motor competence after the first pandemic wave.

As far as we know, our study was the first to report the effects of the pandemic on children in this age group, in which we assessed global and fine motor skills and found that these indicated opposite results. However, it makes sense, since the time of isolation and confinement caused by the pandemic protocols provided more time to play video games [14,24], even though this practise became common even before the pandemic. As a result, fine motor skills increased, serving as an even greater warning for the future of these children.

The main limitations of this study were the small sample and the fact that it was limited to a small geographical area, so it is not possible to objectively state that these results are only a consequence of the pandemic protocols; the fact that there was no daily characterisation of the children’s activities and daily routines may have influenced some of the results, as well as the fact that, although all the children belonged to urban areas, not all of them had the same family and parental involvement, which clearly also has an impact on the development process.

In future studies, we suggest replicating this study with other samples. However, we realise this would be difficult as the data would have had to have been collected before the pandemic. But, following on from the same theme, it would also be interesting to analyse and compare how global and fine motor skill profiles were in studies carried out before the pandemic, compared to studies carried out more recently but with children who had already gone through this process. Another suggestion would be to understand and compare the environment in which the children lived during this period and whether there were any differences in motor development.

## 4. Conclusions

In our study, we demonstrated that the COVID-19 pandemic protocols clearly affected global development. Activities such as running, jumping, kicking, and throwing seemed to be compromised, and we believe that our research can serve as a warning to institutions, early childhood educators, paediatricians, and sports technicians, for the urgency of intervening with activities and physical activity programs for children who at one year of age were subject to confinement.

Given the characteristic negative changes in the motor development of the children evaluated, our results justify the need to closely monitor the development of the pandemic generation and take systematic corrective measures.

## Figures and Tables

**Table 1:** Differences in Motor Skills between Pre-COVID-19 and Post-COVID-19.

PDMS-2	COVID-19	N	M ± SD	*p*	Effect Size
Postural skills (PS)	pre-COVID-19	88	**10.75 ± 0.58**	0.000 *	≥2.0
	post-COVID-19		8.27 ± 0.98		
Locomotion skills (LS)	pre-COVID-19	88	**8.53 ± 0.71**	0.000 *	1.29
	post-COVID-19		7.44 ± 0.82		
Object manipulation skills (OMS)	pre-COVID-19	88	**11.27 ± 1.87**	0.000 *	≥2.0
	post-COVID-19		7.15 ± 1.96		
Fine motor skills (FMS)	pre-COVID-19	88	**8.72 ± 1.24**	0.000 *	1.24
	post-COVID-19		9.37 ± 1.53		
Visuo-motor integration skills (VMIS)	pre-COVID-19	88	10.97 ± 1.53	0.006	0.97
	post-COVID-19		**11.97 ± 1.19**		

* *p* ≤ 0.05 using the Wilcoxon test; significant *p*-values and their associated effects are in bold. N—number of subjects; M—mean of standard scores; SD—standard deviation.

## Data Availability

The data presented in this study are available on request from the corresponding author due to data protection restrictions for minors.
